# Olink proteomics profiling platform reveals non-invasive inflammatory related protein biomarkers in autism spectrum disorder

**DOI:** 10.3389/fnmol.2023.1185021

**Published:** 2023-05-24

**Authors:** Xiao-Hong Bao, Bao-Fu Chen, Jun Liu, Yu-Hua Tan, Shu Chen, Fan Zhang, Hong-Sheng Lu, Ji-Cheng Li

**Affiliations:** ^1^Precision Medicine Center and Department of Cardiothoracic Surgery, Taizhou Central Hospital (Taizhou University Hospital), Taizhou University, Taizhou, China; ^2^Medical Research Center, Yue Bei People's Hospital, Shantou University Medical College, Shaoguan, China; ^3^Department of Children Rehabilitation, Shaoguan Maternal and Child Health Hospital, Shaoguan, China; ^4^Department of Histology and Embryology, School of Basic Medical Sciences, Henan University, Kaifeng, China; ^5^Institute of Cell Biology, Zhejiang University Medical School, Hangzhou, China

**Keywords:** autism spectrum disorder, biomarkers, inflammation, proteomic, Olink

## Abstract

**Background:**

Owing to the lack of valid biomarkers, the diagnosis of autism spectrum disorder (ASD) diagnosis relies solely on the behavioral phenotypes of children. Several researchers have suggested an association between ASD and inflammation; however, the complex relationship between the two is unelucidated to date. Therefore, the current study aims to comprehensively identify novel circulating ASD inflammatory biomarkers.

**Methods:**

Olink proteomics was applied to compare the plasma inflammation-related protein changes in a group of the healthy children (HC, *n* = 33) and another with ASD (*n* = 31). The areas under the receiver operating characteristic curves (AUCs) of the differentially expressed proteins (DEPs) were calculated. The functional analysis of the DEPs was performed using Gene Ontology and Kyoto Encyclopedia Genes and Genomes. Pearson correlation tests were used employed to analyze the correlation between the DEPs and clinical features.

**Results:**

A total of 13 DEPs were significantly up-regulated in the ASD group compared with the HC group. The four proteins, namely, STAMBP, ST1A1, SIRT2, and MMP-10 demonstrated good diagnostic accuracy with the corresponding AUCs (95% confidence interval, CI) of 0.7218 (0.5946–0.8489), 0.7107 (0.5827–0.8387), 0.7016 (0.5713–0.8319), and 0.7006 (0.568–0.8332). Each panel of STAMBP and any other differential protein demonstrated a better classification performance [AUC values from 0.7147 (0.5858–0.8436, STAMBP/AXIN1) to 0.7681 (0.6496–0.8867, STAMBP/MMP-10)]. These DEP profiles were enriched in immune and inflammatory response pathways, including TNF and NOD-like receptor signaling pathways. The interaction between STAMBP and SIRT2 (*R* = 0.97, *p* = 8.52 × 10^−39^) was found to be the most significant. In addition, several DEPs related to clinical features in patients with ASD, particularly AXIN1 (*R* = 0.36, *p* = 0.006), SIRT2 (*R* = 0.34, *p* = 0.010) and STAMBP (*R* = 0.34, *p* = 0.010), were positively correlated with age and parity, indicating that older age and higher parity may be the inflammation-related clinical factors in ASD.

**Conclusion:**

Inflammation plays a crucial role in ASD, and the up-regulated inflammatory proteins may serve as potential early diagnostic biomarkers for ASD.

## 1. Introduction

Autism spectrum disorder (ASD) is a common, highly heritable, and clinically heterogeneous neurodevelopmental disorder characterized by altered social interaction and communication, repetitive behaviors, abnormal sensory experiences, and varying degrees of intellectual disability (Lord et al., 2018, 2020). In addition to aforementioned the core symptoms, several accompanying symptoms such as attention-deficit hyperactivity disorder (ADHD), anxiety, depression, and epilepsy are also common (Lord et al., 2020). From 2012 to 2021, the global prevalence of ASD was 100/10,000 (range 1.09/10,000, 000–436.0/10,000), that is, about 1/100 children were diagnosed with ASD, with more male patients than female patients, and the male-to-female ratio ranged from 0.8 to6.1 (median: 4.2) (Zeidan et al., 2022). The increasing trend of ASD prevalence over time results in increased costs to patients and their families and society, and therefore, breakthroughs in the precise diagnosis and effective treatment of ASD are urgently needed.

The current diagnosis of ASD is based on behavioral assessments, such as the Diagnostic and Statistical Manual of Mental Disorders, Fifth Edition (DSM-5) criteria, and the confirmation of the diagnosis relies on validated observation tools, such as the Childhood Autism Rating Scale, Second Edition (CARS-2) and Diagnostic Observation Schedule for Autism, Second Edition (ADOS-2) (Hyman et al., 2020; Jurek et al., 2022; Kalb et al., 2022). However, the behavior-based diagnosis primarily exhibits the following shortcomings: 1. It can only be evaluated and judged when the behavioral characteristics of ASD occur, while the assessment of the behavior of infants and young children is difficult; 2. The dependence on the subjective judgment of doctors and parents implies, primarily depending on the level of professionalism of doctors and the ability of parents to observe their child’s behavior. This is highly variable and subjective. Early diagnosis and timely intervention are extremely important to reduce the severity of ASD symptoms and improve prognosis (Whitehouse et al., 2021; Singhi and Malhi, 2022). Deficits in behavioral diagnosis tend to delay the exact timing of diagnosis, leading to missed periods of optimal treatment. Biomarkers for early diagnosis can accurately distinguish highly heterogeneous disease groups from controls (Lombardo et al., 2019). However, researchers have not been able to isolate valid and reliable biomarkers for ASD to date (Müller and Amaral, 2017). Therefore, the development of effective biomarkers for early diagnosis of ASD is urgently necessary.

The rapid development of high-throughput omics technologies provides new avenues for dissecting pathophysiological mechanisms and discovering biomarkers in complex diseases. Proteomics is a systems biology approach for studying a group of proteins produced in cells, tissues, and body fluids. The aberrant expression of proteins in a disease reflects both abnormalities in the upstream DNA or RNA molecules and the effects of external stimuli (Mesleh et al., 2021). Thus, measurable protein biomarkers are used for disease diagnosis or to indicate disease severity. Blood is the most widely utilized diagnostic sample due to its low invasiveness. The plasma proteome can originate from any organ or cell and can even exchange between mother and child through the placenta (Pernemalm et al., 2019; Suhre et al., 2021), and plays a crucial role in various biological processes including signal transmission, transport, growth, repair, and defense against infection (Sun et al., 2018). Plasma proteins may develop into an ideal screening library for diagnostic biomarkers because they are frequently dysregulated in several disorders.

Herein, the Olink proteomics platform was used, which employs proximity extension assay (PEA) and bioinformatic technology to analyze the changes in plasma inflammation-related proteins in children with ASD diagnosed by behavioral criteria, investigate plasma novel diagnostic biomarkers, explore the role and possible mechanism of inflammation in the development of ASD, and offers a reference for the early accurate molecular diagnosis of ASD.

## 2. Materials and methods

### 2.1. Study design and participants

**Figure 1:**
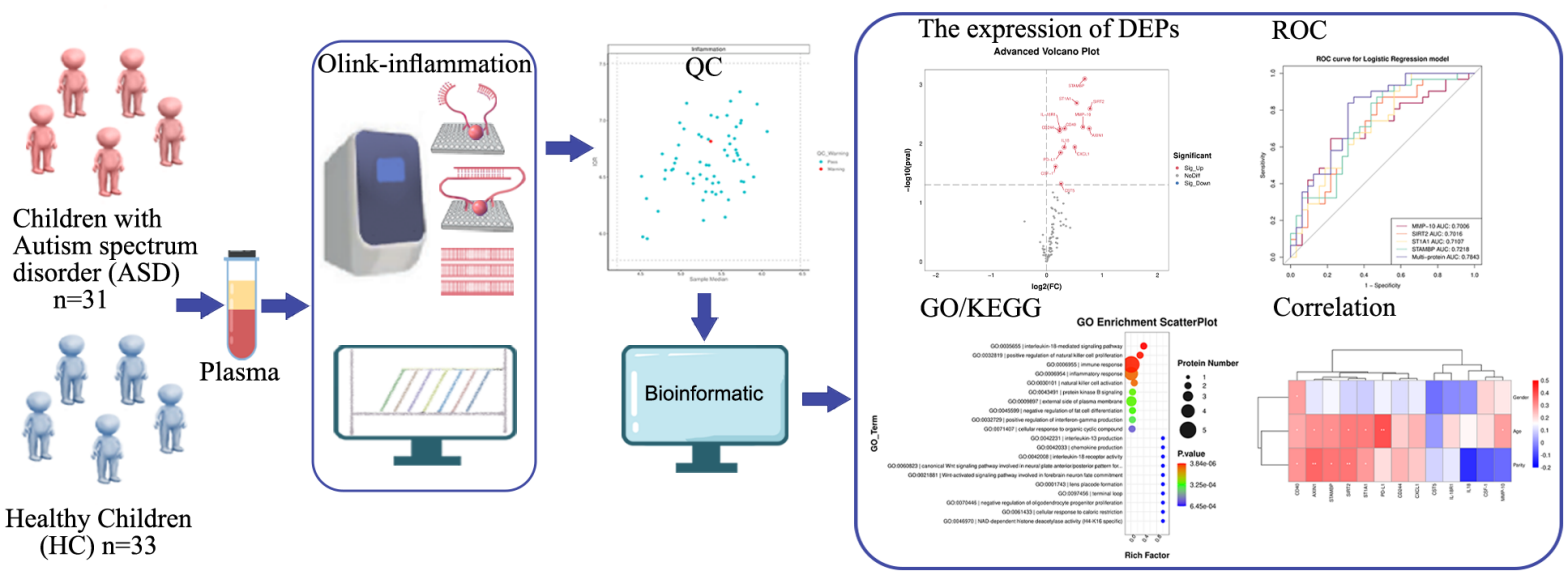
Study strategy and schematic illustration of plasma Olink proteomics. Plasma collected from children with ASD (*n* = 31) and HC (*n =* 33) was used for Olink-inflammation panel. 63 samples passed quality control. Subsequent bioinformatics analysis was performed to evaluate DEPs, diagnostic performance of DEPs (ROC curve), GO/KEGG term enrichment analyses, and correlation of DEPs with various clinical features. ASD, autism spectrum disorder; HC, healthy control; QC, quality control; DEPs, differentially expressed proteins; ROC, receiver operating characteristic; GO, gene ontology; KEGG, kyoto encyclopedia genes and genomes.

A total of 31patients with ASD and 33 healthy controls (HC) were enrolled between February 2021 and November 2022 at the Shaoguan Maternal and Child Health Care Hospital for the current study. The plasma proteomics of the ASD and HC groups were identified and compared (Figure 1). The children in the ASD group were diagnosed with ASD through the DSM-5 diagnostic criteria. The clinicians in pediatric rehabilitation assess children’s symptoms with the CARS-2 score. The exclusion criteria were as follows: children suffering from schizophrenia; children with pure mental developmental disorders; children with simple language developmental disorders; children with other pervasive developmental disorders, deafness, and organic diseases of the nervous system; children suffering from diseases of heart, liver, and kidney; and patients who have inflammation or infectious diseases and are taking drugs during the study.

### 2.2. Sample collection

Peripheral venous blood (3 mL) was collected from each patient into EDTA tubes in the morning after undergoing overnight fasting to eliminate diet-induced variations. Immediately after blood collection, plasma was obtained by centrifugation (3,000 rpm for 15 min at 4°C) and stored at −80°C until laboratory analysis.

### 2.3. Proteomic analysis

Plasma inflammatory markers were assessed using the commercially available Olink® Target 96 Inflammation panels from Olink (Uppsala, Sweden).^1^ In brief, the target protein binds to the double oligonucleotide-labeled antibody probe with high specificity, and then the microfluidic real-time PCR amplification of the oligonucleotide sequence is used to quantitatively detect the resulting DNA sequence. Using internal and external controls, the resulting threshold cycle (Ct)-data were processed for quality control and normalized. Normalized Protein expression (NPX) values were provided as the final assay read-out, which was an arbitrary log2-scale unit corresponding to higher protein levels. One control sample that failed to pass the quality control, was excluded from further analysis.

### 2.4. DEP analysis

The R package “Olink®Analyze” was used to identify the sets of DEPs between the two groups. Proteins with a *p*-value of <0.05 were considered to be differentially expressed. The visualization of DEPs including volcano plots and heatmaps was performed using the standard R package “ggplot2.”

The diagnostic performance of DEPs was assessed by receiver operating characteristic (ROC) curves. AUC was recorded as an index of diagnostic accuracy and compared among the proteins. According to logistic regression, two or more indicators were combined, and the AUC value of the combined diagnosis is shown in the figure legend at the lower right corner so that the combined diagnostic effect can be viewed. A higher AUC value reflected the greater performance of the classifier. The AUC value of 1.0 represented a perfect assignment, whereas an AUC of 0.5 represented an unreliable test (gray line).

### 2.5. Go enrichment analysis and pathway enrichment analysis

All GO terms that were significantly enriched in DEPs compared to the genome background are provided by GO enrichment analysis (Sun et al., 2018). In addition, the signaling pathway enrichment analysis identified significantly enriched metabolic pathways or signal transduction pathways in DEPs compared with the whole genome background (Kanehisa et al., 2021). GO and KEGG analyses were performed by cluster Profiler using the R software package. The “ggplot2” R tool was used to visualize the findings of GO and KEGG enrichment analysis, and the top 20 GO terms and KEGG pathways were shown as a bubble chart.

### 2.6. Correlation analysis

Pearson’s correlation analysis was used to determine the correlation between the expression levels of two DEPs, and the scatterplots illustrated the strongest correlation. Pearson correlation tests were also employed to analyze the correlation between the DEPs and clinical features of patients. The closer the correlation coefficient gets to 1, the better the correlation between the two variables. The significance of correlation coefficients was calculated using the *p*-value calculator for correlation coefficients.

### 2.7. Statistical analysis

All statistical analyses were performed using the R software “Olink®Analyze” (V.2.0.0). A value of *p* of less than 0.05 was considered statistically significant.

## 3. Results

### 3.1. Characteristics of the study subjects

Olink proteomic analysis of plasma from children with ASD diagnosed by CARS score and plasma from normal children was performed to identify potential diagnostic biomarkers of ASD (Table 1). A total of 64 samples were included in this cohort (31 ASD *VS* 33 HC). The majority of ASD patients were male (80.65%), and the proportion of children with more than second birth was higher than that of first birth (60.71%: 39.29%). Based on the CARS score, it can be concluded that the number of patients with mild ASD was higher than those with moderate or severe ASD (74.19%: 25.81%).

**Table 1:** Demographics of clinical samples analyzed by Olink.

Characteristics	ASD (*n* = 31)	HC (*n* = 33)	*P* Values^a^
Gender (Male/Female)	25/6	18/15	0.026 (Chi-square test)
Age (years), median (IQR)	4 (2.8333, 4.4583)	3.4167 (3.0833, 5)	0.877 (Wilcoxon test)
Parity, *n* (%)			0.672 (Chi-square test)
1	11 (39.29)	13 (44.83)	
≥2	17 (60.71)	16 (55.17)	
CARS Score, *n* (%)			
Mild (30–36)	23 (74.19)		
Moderate or severe (≥37)	8 (25.81)		

a *P* values was calculated using Chi-square test (Gender and Parity) and Wilcoxon test (Age). ASD, autism spectrum disorder; HC, healthy control; IQR, interquartile range; CARS, childhood autism rating scale, second edition.

### 3.2. Up-regulation of inflammation-related proteins in ASD plasma

**Figure 2:**
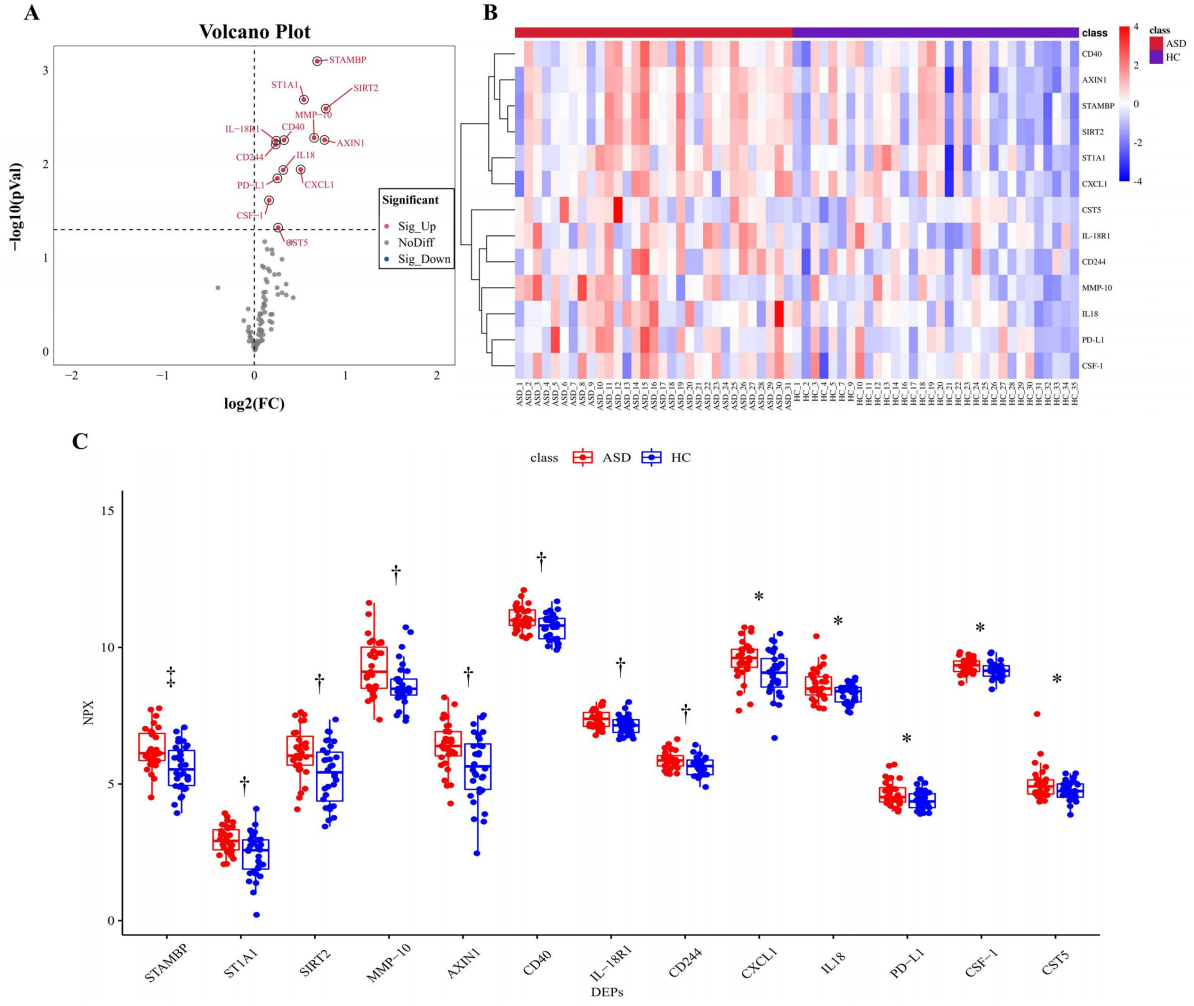
All significantly changed inflammation-related proteins between the ASD group and the HC group. **(A)** Volcanic visualization of 92 inflammation-related biomarkers. Red, significantly up-regulated proteins. Gray, no differences. Blue, significantly down-regulated proteins. **(B)** Heatmap of differentially expressed inflammation-related proteins. **(C)** Box-scatter plot of the 13 DEPs expression. ^*^p ≤ 0.05, ^†^*p* ≤ 0.01, ^‡^*p* ≤ 0.001. ASD, autism spectrum disorder; HC, healthy control; NPX, normalized protein expression; DEPs, differentially expressed proteins.

Olink inflammation panel was employed to detect differences in the expression of inflammation-related proteins in ASD and HC samples (See Supplementary Table S1 for details of inflammation panel). A total of 13 proteins were significantly up-regulated in the ASD group compared with the HC group (Figure 2A; Table 2), including STAMBP, ST1A1, SIRT2, MMP-10, AXIN1, CD40, IL-18R1, CD244, CXCL1, IL18, PD-L1, CSF-1, and CST5. The expression heatmap of the above DEPs in each sample was shown in Figure 2B. The difference in each of DEPs between the ASD and the HC groups was significant (Figure 2C). However, the difference in STAMBP was the most significant (*p* = 0.0008).

**Table 2:** Significantly changed plasma inflammatory proteins between the ASD group and the HC group.

Protein symbol	Uniprot ID	Name	FC^a^	*p* values^b^
STAMBP	O95630	STAM-binding protein	0.69	0.0008
ST1A1	P50225	Sulfotransferase 1A1	0.54	0.0021
SIRT2	Q8IXJ6	NAD-dependent protein deacetylase sirtuin-2	0.78	0.0026
MMP-10	P09238	Matrix metalloproteinase-10	0.65	0.0053
AXIN1	O15169	Axin-1	0.77	0.0055
CD40	P25942	Tumor necrosis factor receptor superfamily member 5	0.33	0.0056
IL-18R1	Q13478	Interleukin-18 receptor 1	0.24	0.0056
CD244	Q9BZW8	Natural killer cell receptor 2B4	0.23	0.0062
CXCL1	P09341	Growth-regulated alpha protein	0.51	0.0114
IL18	Q14116	Interleukin-18	0.32	0.0116
PD-L1	Q9NZQ7	Programmed cell death 1 ligand 1	0.25	0.0142
CSF-1	P09603	Macrophage colony-stimulating factor 1	0.16	0.0244
CST5	P28325	Cystatin-D	0.26	0.0476

^a^FC (Fold-change) between ASD and HC, calculated on a log2 scale. ^b^*p* values calculated using Student’s *t*-test. Significantly different proteins are shown (*p* < 0.05).

### 3.3. Important diagnostic values of DEPs

**Figure 3:**
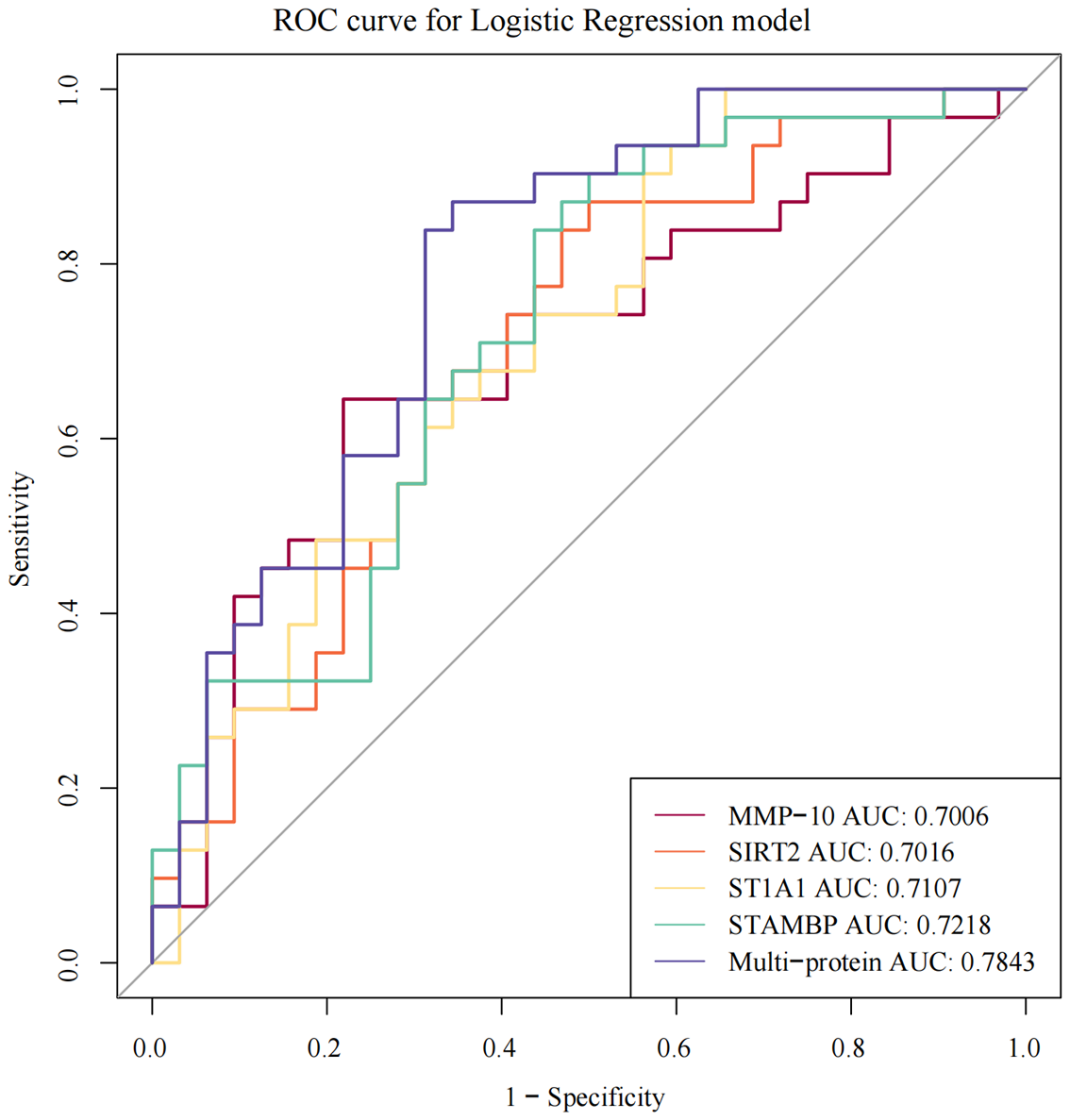
The ROC curves of individual and multi-protein with AUC > 0.7 showing sensitivity and specificity of DEPs in ASD. ROC receiver operating characteristic, AUC the area under the receiver operating characteristic curves.

The ROCs were plotted based on both the true and false positive rates, and the AUCs of 13 DEPs were calculated. The following four proteins had AUC (95% CI) values greater than 0.7: STAMBP, ST1A1, SIRT2, and MMP-10, the actual values of being 0.7218 (0.5946–0.8489), 0.7107 (0.5827–0.8387), 0.7016 (0.5713–0.8319), and 0.7006 (0.568–0.8332), respectively (Figure 3). Among them, STAMBP had the highest AUC and its diagnostic value was superior to the other three DEPs. The logistic regression model was also computed with the overall ROC curves for the aforementioned four DEPs. A classifier consisting of these four DEPs was higher than a single STAMBP, and the AUC increased from 0.7218 (0.5946–0.8489) to 0.7843 (0.67–0.8985). The AUC values for all DEPs are listed in Supplementary Table S2. However, when the AUCs for the individual DEPs in combination with STAMBP were considered, MMP-10 performed better than other DEPs (Supplementary Table S2).

### 3.4. DEPs enrichment analysis and correlation analysis

**Figure 4:**
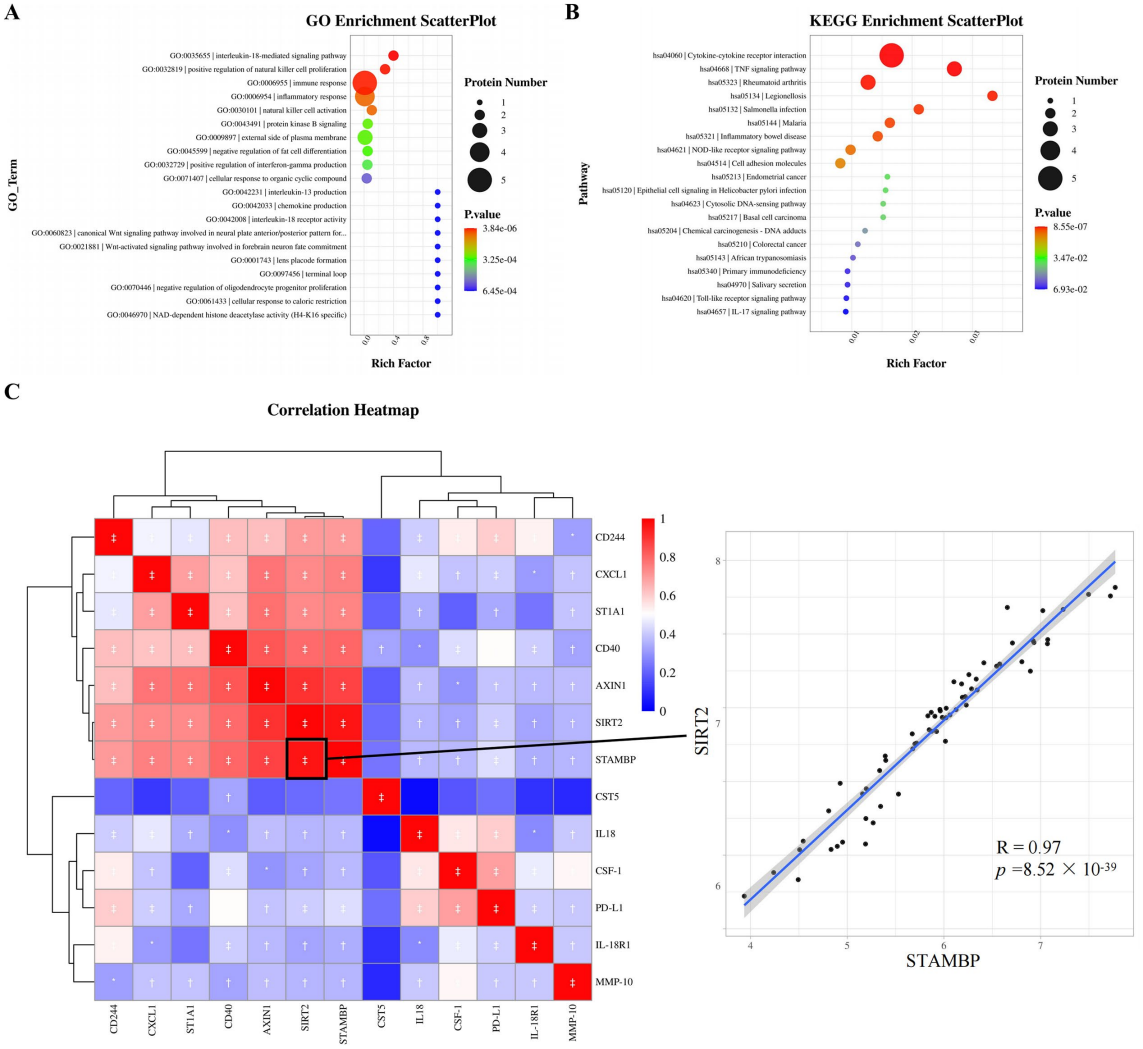
Functional enrichment analysis and correlation analysis of DEPs. **(A)** Top 20 enriched GO terms based on the background of all annotated proteins. **(B)** Top twenty enriched KEGG pathways based on the background of all annotated proteins. **(C)** Correlations between DEPs in ASD and HC. Red, positively related; blue, negatively related; and white, nonrelated. The scatterplot shows the highest correlation between STAMBP and SIRT2. ^*^p ≤ 0.05, ^†^*p* ≤ 0.01, ^‡^*p* ≤ 0.001. GO, gene ontology; KEGG, kyoto encyclopedia genes and genomes, R Pearson correlation coefficient.

GO and KEGG enrichment analysis was used to further investigate the function of plasma DEPs in ASD. DEPs were mainly enriched in the GO terms such as interleukin-18-mediated signaling pathway, positive regulation of natural cell proliferation, immune response, and inflammatory response (Figure 4A). KEGG pathways such as cytokine-cytokine receptor interaction, TNF signaling pathway, and NOD-like receptor signaling pathway were significantly enriched (Figure 4B). The protein expression correlations between different DEPs were analyzed based on the NPX values. All 13 DEGs were upregulated proteins, and hence correlations between the DEPs demonstrated positive correlations. Strong correlations were revealed between STAMBP and SIRT2 (*R* = 0.97, *p* = 8.52 × 10^−39^), SIRT2 and AXIN1 (*R* = 0.91, *p* = 1.76 × 10^−24^), STAMBP and AXIN1 (*R* = 0.88, *p* = 4.15 × 10^−21^), and AXIN1 and CD40 (*R* = 0.83, *p* = 2.86 × 10^−17^) proteins (Figure 4C), with the STAMBP and SIRT2 expressions having the strongest correlation in plasma.

### 3.5. Correlation analysis between differential proteins and clinical characteristics

**Figure 5:**
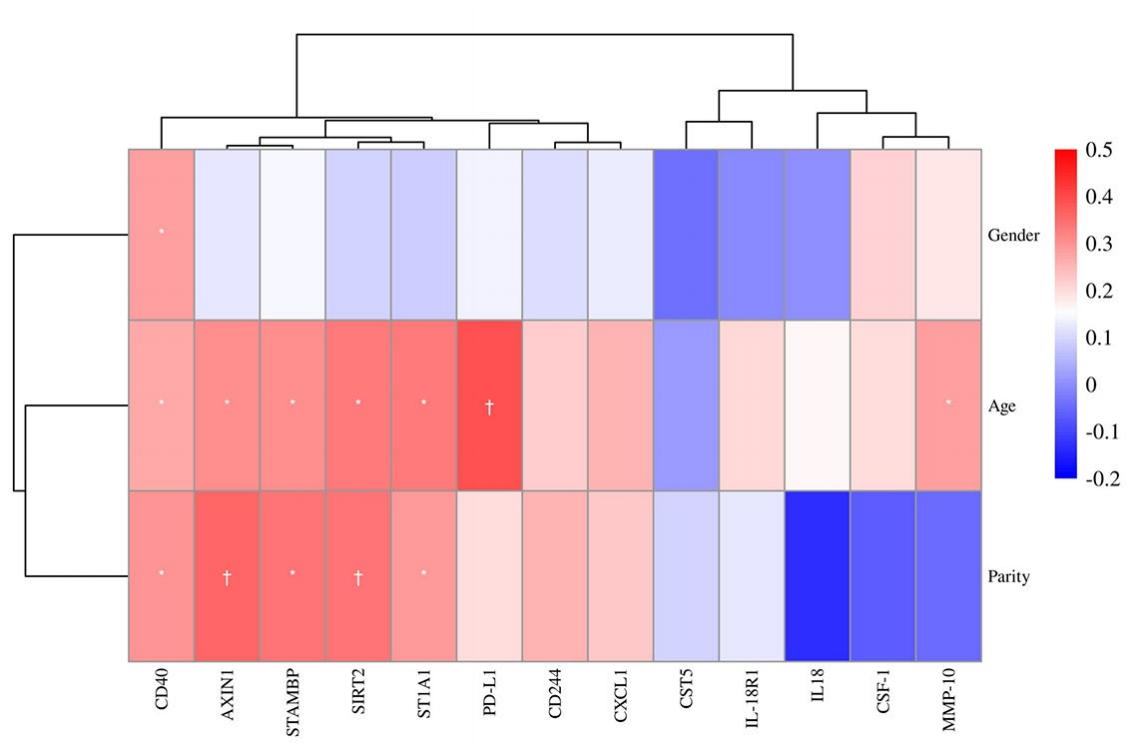
Correlation heatmap of gender, age and parity among the protein expressions of 13 DEPs. Red, positively related; blue, negatively related; and white, nonrelated. ^*^p ≤ 0.05, ^†^*p* ≤ 0.01.

Next, the association between plasma DEPs and clinical characteristics was analyzed (Figure 5). Five proteins, PD-L1 (*R* = 0.39, *p* = 0.003), ST1A1 (*R* = 0.34, *p* = 0.011), SIRT2 (*R* = 0.33, *p* = 0.013), STAMBP (*R* = 0.31, *p* = 0.022), and AXIN1 (*R* = 0.30, *p* = 0.023), were positively associated with child age. Three candidate markers, AXIN1 (*R* = 0.36, *p* = 0.006), SIRT2 (*R* = 0.34, *p* = 0.010), and STAMBP (*R* = 0.34, *p* = 0.010), were in turn positively associated with parity. In addition, these candidate DEPs were found to be independent of gender.

## 4. Discussion

The clinical heterogeneity and complex etiology and pathogenesis of ASD make the precise diagnosis of ASD challenging. Changes in societal awareness and diagnostic criteria can also affect the rates of ASD diagnosis. A recent study reporting the incidence and prevalence of ASD across 204 countries indicated that in the past 30 years (1990–2019), the number of people with ASD increased from an estimated 20 million [95% uncertainty level (UI) =16.9–24.2)]to more over 28 million (95% UI = 23.5–33.8), with a relative increase in the prevalence of ASD of 39.3% worldwide (Solmi et al., 2022). The prevalence of ASD varies by country or region. Currently, Iceland has the highest incidence of ASD worldwide, with a prevalence of 3.13% (3,130/100,000) in children aged 7–9 years (Weye et al., 2021). ASD prevalence in children aged 6–12 years in China was 0.70% (95% CI: 0.64–0.74%, 1 in 143) according to the first national ASD prevalence statistics (Zhou et al., 2020), which was lower than 1.85% (95% CI: 18.0–19.1, 1 in 54) in the children aged 8 years in the United States (Maenner et al., 2020) and 2.5% (95% CI: 2.0–3.0) in children aged 6–7 years in Australia (Randall et al., 2016). The low prevalence of ASD in Chinese children may be caused by the parents’ educational level as well as insufficient awareness of ASD, and the use of diagnostic criteria DSM-5 under equivalent conditions that may reduce the prevalence. The male-to-female ratio in patients screened for inclusion in our study was approximately 4:1, which is comparable to the data from previous findings (Zeidan et al., 2022). For people with ASD, the number of males is greater than that of females, and the proportion of the former has remained above >3 for the past 30 years. However, the male-to-female ratio gradually declined from 1990 to 2019, possibly because of the increased clinical concern for female ASD patients (Solmi et al., 2022). In China, the prevalence was also significantly higher in boys than in girls (0.95% vs. 0.30% (Zhou et al., 2020); 0.7277% vs. 0.1645% (Wang et al., 2018). However, the molecular mechanism of this “preference” for males is unelucidated to date. It may be related to mutations in X-linked genes encoding the neural junction proteins NLGN3 and NLGN4 (Jamain et al., 2003; Nguyen et al., 2020). Alternatively, it may be caused by pathogenic variants that alter the function of the CDK16 and TRPC5 genes on the X chromosome (Leitão et al., 2022).

In recent years, several studies have found an association between inflammation and ASD (Croonenberghs et al., 2002; Siniscalco et al., 2018). A systematic review investigated the pro-inflammatory markers in approximately 4,000 children and adolescents with neuropsychiatric and neurodevelopmental disorders, including ASD. The findings of this review indicated that the inflammatory markers were elevated, more significantly in ASD, thus identifying the role of inflammation in these neuropsychiatric disorders and providing preliminary evidence linking pro-inflammatory status with the disorders (Mitchell and Goldstein, 2014). According to inflammation-related cytokines produced by the lipopolysaccharide stimulation of cultured peripheral blood mononuclear cells, ASD can be divided into pro-inflammatory and non-inflammatory groups., Children with pro-inflammatory features demonstrate more severely impaired development, higher behavioral scores, and more prominent sleep problems and aggressive behaviors compared to those with non-inflammatory features (Careaga et al., 2017). An increase in pro-inflammatory biomarkers in blood, such as various interleukins, reinforce the strong association of abnormal inflammatory response with ASD (Siniscalco et al., 2018). Therefore, inflammation-related proteins are likely to be a high-quality screening library for diagnostic biomarkers in ASD. However, the majority of studies on ASD have focused on several or at most a dozen inflammatory factors without providing a more comprehensive analysis of the inflammation-related proteins.

In the current study, the plasma inflammation-related protein profiles were comprehensively compared among 31 children with ASD and 33 healthy controls by Olink proteomics, and 13 significantly up-regulated DEPs were identified. The DEPs were enriched in inflammation and immune response, and were significantly related to several immune-related pathways such as the TNF signaling pathway. Four proteins including STAMBP, ST1A1, SIRT2 and MMP-10 demonstrated good diagnostic accuracy, and the combination of STAMBP with any of the differential proteins exhibited good classification performance. The findings of the analysis also revealed that there were strong correlations between four protein combinations: STAMBP and SIRT2, SIRT2 and AXIN1, STAMBP and AXIN1, and AXIN1 and CD40. Several ASD DEPs were positively correlated with age and parity. In addition, these inflammatory differential proteins were not significantly associated with gender, indicating that inflammatory status did not differ between male and female patients.

The levels of the inflammatory proteins such as interleukin-18 (IL-18), chemokine (CXCL1), macrophage colony-stimulating factor (CSF-1), cytokine receptors (IL-18R1 and CD40), ligands (CD244 and PD-L1), and other inflammation-related markers (STAMBP, ST1A1, SIRT2, MMP-10, AXIN1, and CST5) were increased in the ASD group. Among them, the up-regulation of IL-18 in ASD was consistent with the results of the previous studies (Businaro et al., 2016; Saresella et al., 2016). The remaining 12 DEPs were novel findings of this study. All DEPs were enriched in some inflammation-related signaling pathways, such as the TNF signaling pathway and NOD-like receptor signaling pathway. A study by Ziats (Ziats and Rennert, 2011) indicated that ASD transmits immune signals primarily through TNF, JNK, and NF-κB. A previous *in vitro* study demonstrated that the TNF-JNK pathway and TNF-p38 MAPK pathway stimulated CXCL1 release from human endothelial cells (Lo et al., 2014). TNF-α signaling was associated with the polarization of macrophages to autoimmune inflammatory states during injury repair, and autocrine TNF-α signaling induced the expression of proteins such as CXCL1 in macrophages to activate and recruit immune cells (Wang et al., 2022). Furthermore, CSF-1 promoted cell proliferation, differentiation, and cytokine expression by binding to the CSF-1R receptor and activating the MAPK signaling pathway (Muñoz-Garcia et al., 2021). The activation of the NOD-like receptor signaling pathway is also one of our novel findings, which may initiate the innate immune response through the NF-κB pathway. However, the detailed mechanisms of these signaling pathways in ASD still need to be investigated in depth.

The AUC values were calculated based on the ROC curves for the DEPs, with the four proteins having the highest diagnostic values (STAMBP, ST1A1, SIRT2, and MMP-10). These proteins may serve as potential diagnostic plasma biomarkers for ASD. STAMBP is a deubiquitinated protein encoded by the p13 STAMBP gene on chromosome 2 (Tanaka et al., 1999), and was found to be significantly differentiated in ASD. The STAMBP protein has been reported as a potential diagnostic biomarker for early Alzheimer ‘s disease (Whelan et al., 2019), late pregnancy in women with postpartum depression (Bränn et al., 2017), fibromyalgia (Fineschi et al., 2022), and esophageal squamous cell carcinoma (Aversa et al., 2020). STAMBP may increase NALP7 (NACHT, LRR and PYD domains-containing protein 7) abundance by inhibiting the trafficking of the inflammasome NALP7 to lysosomes and preventing the lysosomal degradation of NALP7 (Bednash et al., 2017). NALP7 regulates innate immune responses by promoting the maturation of the inflammatory cytokines IL-1β and IL-18. ST1A1 (SULT1A1) is a sulfotransferase that catalyzes the sulfation of catecholamines, estrogens, phenolics, and neurotransmitters and plays a crucial role in phase II drug metabolism (Isvoran et al., 2022). ST1A1, like STAMBP, is down-regulated during late pregnancy in women with postpartum depression (Bränn et al., 2017) and is associated with an increased risk of esophageal squamous cell carcinoma (Aversa et al., 2020). Furthermore, changes in ST1A1 expression may also cause inflammation in the skin lesions of patients with cutaneous leishmaniasis (Taslimi et al., 2020) and predict short-term mortality in patients with acute myocardial infarction (Schmitz et al., 2022). SIRT2 is the only NAD-dependent deacetylase primarily localized in the cytoplasm, which plays an important regulatory role in biological processes such as neural cell differentiation and survival, mitotic regulation, genomic integrity, cell differentiation, cell homeostasis, aging, infection, inflammation, oxidative stress, and autophagy (Wang et al., 2019). Abnormality in SIRT2 blood content in various diseases may indicate that SIRT2 is probably an effective biomarker for the diagnosis and treatment of these diseases (Hysing et al., 2019; Panezai et al., 2020; Qu et al., 2021; Zhu et al., 2021; Fineschi et al., 2022). STAMBP demonstrated the highest correlation with SIRT2 in ASD (*R* = 0.97, *p* = 8.52 × 10^−39^). A possible reason for this may be that NACHT, LRR and PYD domains-containing protein 3 (NLRP3) inflammasome was significantly upregulated in children with ASD (Saresella et al., 2016), whereas both STAMBP (Bednash et al., 2021) and SIRT2 (He et al., 2020) may participate in the inflammatory response by regulating NLRP3 inflammasome. Furthermore, MMP-10 expression was significantly higher in the ASD group compared with the normal controls. MMP-10 promotes the recruitment of infiltrating cells by remodeling the extracellular matrix (Pittayapruek et al., 2016). The up-regulation of MMP-10 has also been shown to be linked to some other neurological diseases, such as Alzheimer ‘s disease (Whelan et al., 2019; Martino Adami et al., 2022), dementia (Erhardt et al., 2021), and intracerebral hemorrhage (Howe et al., 2018).

**Figure 6:**
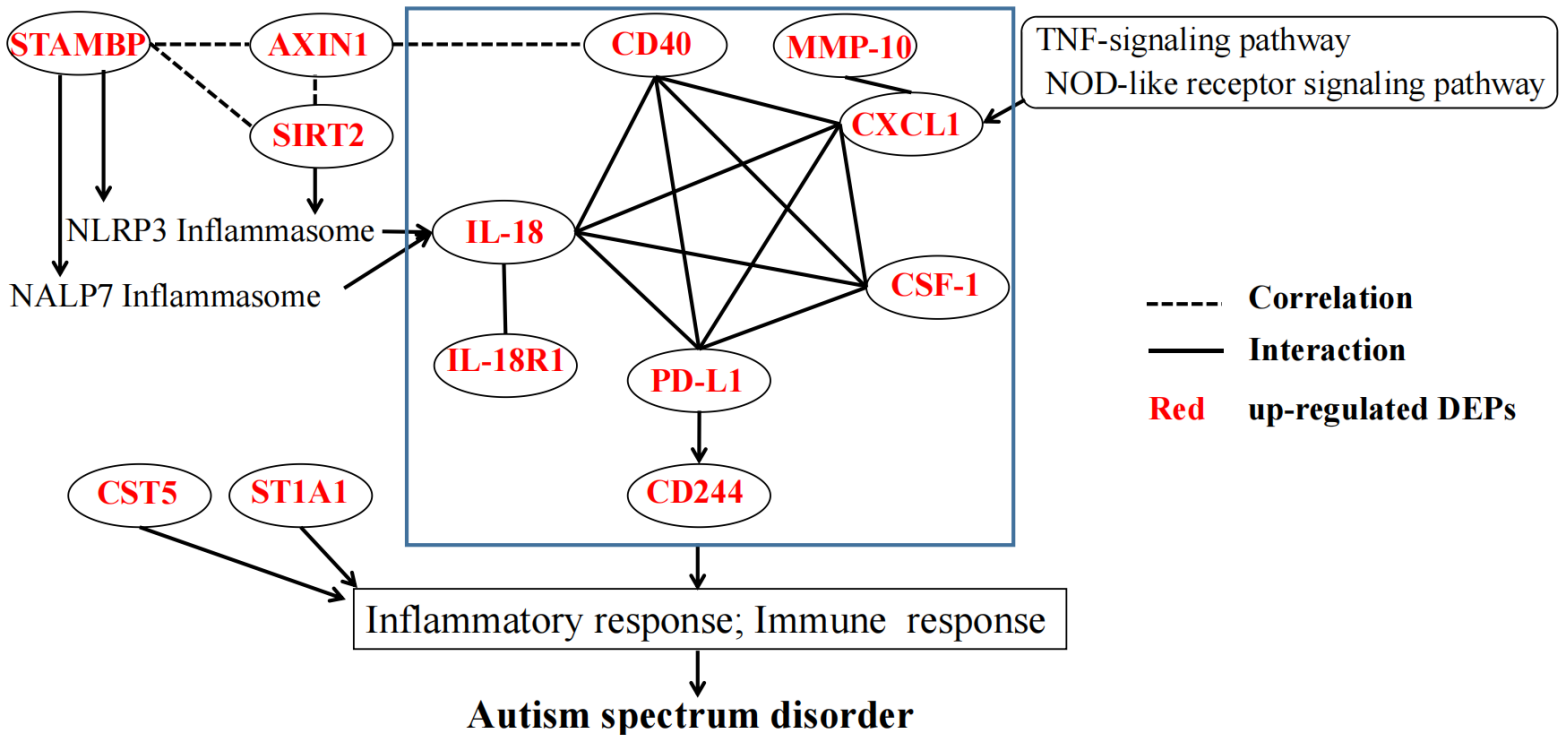
The potential mechanism linking DEPs in the pathogenesis of ASD. The blue rectangle indicates STRING protein interaction analysis of partial DEPs. STAMBP, STAM-binding protein; ST1A1, Sulfotransferase 1A1; SIRT2, NAD-dependent protein deacetylase sirtuin-2; MMP-10, Matrix metalloproteinase-10; AXIN1, Axin-1; CD40, Tumor necrosis factor receptor superfamily member 5; IL-18R1, Interleukin-18 receptor 1; CD244, Natural killer cell receptor 2B4; CXCL1, Growth-regulated alpha protein; IL18, Interleukin-18; PD-L1, Programmed cell death 1 ligand 1; CSF-1, Macrophage colony-stimulating factor 1, CST5 Cystatin-D, NLRP3 NACHT, LRR and PYD domains-containing protein 3, NALP7 NACHT, LRR and PYD domains-containing protein 7.

Based on the aforementioned reported literature, inflammatory DEPs may induce the inflammatory or immune response through a very complex mechanism, thereby contributing to ASD development (Figure 6). STRING protein interaction analysis^2^ revealed complex interactions among eight DEPs (CD40, CXCL1, IL-18, IL-18R1, CSF-1, PD-L1, CD244 and MMP-10). However, STAMBP and SIRT2 may promote IL-18 and inflammatory response through the inflammasomes NALP7 and NLRP3. The interaction between SATMBP, SIRT2, AXIN1 and CD40 have not yet been reported, but they must be linked by some mechanism. In addition, the TNF and NOD signaling pathway may play an important role in the pathogenesis of ASD through CXCL1 or IL18.

Molecular expression profiles in ASD vary across age groups (Ramsey et al., 2013). Correlation analysis between DEPs and age in ASD revealed that five inflammatory proteins (PD-L1, ST1A1, SIRT2, STAMBP, and AXIN1) were positively associated with age at diagnosis. Ramsey and colleagues (Ramsey et al., 2013) also discovered some age-related proteins in ASD, but they were different from the ones found in the current study. One major reason is that diverse proteomic platforms detect different proteins. Using a highly specific PEA technique that focused on the detection of inflammatory biomarker profiles can be a technical advantage in this study. Positive correlations between AXIN1, SIRT2, and STAMBP and parity were also found. Although it had been pointed out that ASD is relevant to prenatal factor–parity (Bilder et al., 2009; Cheslack-Postava et al., 2014), little is known about the association between inflammatory markers in ASD and parity. Consequently, clinical cohort studies with rational design need to be performed to further assess the association of clinical factors such as age and parity with inflammation in ASD.

A few limitations of this study should be mentioned. First, the number of participants was small and the validation of the candidate biomarker in an independent cohort was lacking. The primary reason is that a large enough sample could not be collected because of the refusal of the child and parents and several technical reasons while collecting blood. Second, this study was a single-center study. The findings of our study must be replicated in other centers so that the inflammatory biomarkers can be widely disseminated for ASD diagnosis. Therefore, a larger, multicenter study is needed to confirm our results. Third, our findings were from a single-layer omic analysis. In comparison to single-layer omic analysis, multi-omics analysis exhibits higher sensitivity (the missing data in one omics analysis can be supplemented with other measurements) and reliability (multiple measurements are more accurate). However, this study directly identified the expression changes of inflammatory proteins at the protein level of ASD and healthy groups, providing useful insight for future multi-omics research. It will be more convincing if follow-up studies combine single-cell transcriptomics and transcriptomics to verify the changes of DEPs in mRNA transcription at the level of single cells or even subcellular structures.

## 5. Conclusion

In summary, 13 inflammation-related DEPs were observed in the plasma of children, and four of them exhibited high diagnostic accuracy and may serve as potential diagnostic biomarkers for ASD. The enrichment of these DEPs in inflammatory and immune-related responses and signaling pathways illustrated the importance of inflammation in the development of ASD. Although the molecular mechanisms through which these biomarker proteins played a role in ASD are unelucidated to date, the findings of this study may provide new avenues for the early diagnosis and monitoring of ASD. In the future, we will expand the clinical sample sizes, improve the diagnostic accuracy of candidate biomarkers, and validate their potential for clinical application.

## Data availability statement

The original contributions presented in the study are included in the article/supplementary material, further inquiries can be directed to the corresponding author/s.

## Ethics statement

The studies involving human participants were reviewed and approved by the Shaoguan Maternal and Child Health Care Hospital, China. Written informed consent to participate in this study was provided by the participants’ legal guardian/next of kin.

## Author contributions

X-HB contributed to data curation, formal analysis, visualization, methodology, and writing of the original draft. B-FC and H-SL contributed to funding acquisition, writing-reviewing, and editing. Y-HT and SC contributed to resources, clinic data curation, and formal analysis. JL contributed to formal analysis, methodology, and visualization. FZ contributed to resources, data curation, and formal analysis. J-CL contributed to conceptualization and design, funding acquisition, project management, resources, supervision, and review and editing. All authors agreed on the final version of the manuscript. All authors contributed to the article and approved the submitted version.

## Funding

This study was financially supported by the Shaoguan City Social Development Technology Collaborative Innovation System Construction Project (grant number: 220602114530885).

## Conflict of interest

The authors declare that the research was conducted in the absence of any commercial or financial relationships that could be construed as a potential conflict of interest.

## Publisher’s note

All claims expressed in this article are solely those of the authors and do not necessarily represent those of their affiliated organizations, or those of the publisher, the editors and the reviewers. Any product that may be evaluated in this article, or claim that may be made by its manufacturer, is not guaranteed or endorsed by the publisher.

## Footnotes

^1^ https://olink.com

^2^string-db.org
