# Supplementary material for: Olink proteomics profiling platform reveals non-invasive inflammatory related protein biomarkers in autism spectrum disorder
Source: Front Mol Neurosci. 2023 May 24;16:1185021. doi: 10.3389/fnmol.2023.1185021 (PMC10244537; doi:10.3389/fnmol.2023.1185021)
Supplement: Supplementary file 1 [file Table_1.docx]

Supplementary Material

Olink Proteomics Profiling Platform Reveals Non-invasive Inflammatory Related Protein Biomarkers in Autism Spectrum Disorder

Xiao-Hong Bao*

*** Correspondence:** Ji-Cheng Li: [zjulijicheng@163.com](mailto:zjulijicheng@163.com)

# Table S1. 96-inflammation panel.

| Protein Symbol | Uniprot ID | Name |
| --- | --- | --- |
| ADA | P00813 | Adenosine Deaminase |
| ARTN | Q5T4W7 | Artemin |
| AXIN1 | O15169 | Axin-1 |
| Beta-NGF | P01138 | Beta-nerve growth factor |
| CASP-8 | Q14790 | Caspase-8 |
| CCL3 | P10147 | C-C motif chemokine 3 |
| CCL4 | P13236 | C-C motif chemokine 4 |
| CCL19 | Q99731 | C-C motif chemokine 19 |
| CCL20 | P78556 | C-C motif chemokine 20 |
| CCL23 | P55773 | C-C motif chemokine 23 |
| CCL25 | O15444 | C-C motif chemokine 25 |
| CCL28 | Q9NRJ3 | C-C motif chemokine 28 |
| CD40 | P25942 | CD40L receptor |
| CDCP1 | Q9H5V8 | CUB domain-containing protein 1 |
| CXCL1 | P09341 | C-X-C motif chemokine 1 |
| CXCL5 | P42830 | C-X-C motif chemokine 5 |
| CXCL6 | P80162 | C-X-C motif chemokine 6 |
| CXCL9 | Q07325 | C-X-C motif chemokine 9 |
| CXCL10 | P02778 | C-X-C motif chemokine 10 |
| CXCL11 | O14625 | C-X-C motif chemokine 11 |
| CST5 | P28325 | Cystatin D |
| DNER | Q8NFT8 | Delta and Notch-like epidermal growth factor-related receptor |
| CCL11 | P51671 | Eotaxin |
| 4E-BP1 | Q13541 | Eukaryotic translation initiation factor 4E-binding protein 1 |
| FGF-21 | Q9NSA1 | Fibroblast growth factor 21 |
| FGF-23 | Q9GZV9 | Fibroblast growth factor 23 |
| FGF-5 | P12034 | Fibroblast growth factor 5 |
| FGF-19 | O95750 | Fibroblast growth factor 19 |
| Flt3L | P49771 | Fms-related tyrosine kinase 3 ligand |
| CX3CL1 | P78423 | Fractalkine |
| GDNF | P39905 | Glial cell line-derived neurotrophic factor |
| HGF | P14210 | Hepatocyte growth factor |
| IFN-gamma | P01579 | Interferon gamma |
| IL-1 alpha | P01583 | Interleukin-1 alpha |
| IL-2 | P60568 | Interleukin-2 |
| IL-2RB | P14784 | Interleukin-2 receptor subunit beta |
| IL-4 | P05112 | Interleukin-4 |
| IL5 | P05113 | Interleukin-5 |
| IL6 | P05231 | Interleukin-6 |
| IL-7 | P13232 | Interleukin-7 |
| IL-8 | P10145 | Interleukin-8 |
| IL10 | P22301 | Interleukin-10 |
| IL-10RA | Q13651 | Interleukin-10 receptor subunit alpha |
| IL-10RB | Q08334 | Interleukin-10 receptor subunit beta |
| IL-12B | P29460 | Interleukin-12 subunit beta |
| IL-13 | P35225 | Interleukin-13 |
| IL-15RA | Q13261 | Interleukin-15 receptor subunit alpha |
| IL-17A | Q16552 | Interleukin-17A |
| IL-17C | Q9P0M4 | Interleukin-17C |
| IL-18 | Q14116 | Interleukin-18 |
| IL-18R1 | Q13478 | Interleukin-18 receptor 1 |
| IL-20 | Q9NYY1 | Interleukin-20 |
| IL-20RA | Q9UHF4 | Interleukin-20 receptor subunit alpha |
| IL-22 RA1 | Q8N6P7 | Interleukin-22 receptor subunit alpha-1 |
| IL-24 | Q13007 | Interleukin-24 |
| IL-33 | O95760 | Interleukin-33 |
| LAP TGF-beta-1 | P01137 | Latency-associated peptide transforming growth factor beta-1 |
| LIF | P15018 | Leukemia inhibitory factor |
| LIF-R | P42702 | Leukemia inhibitory factor receptor |
| CSF-1 | P09603 | Macrophage colony-stimulating factor 1 |
| MMP-1 | P03956 | Matrix metalloproteinase-1 |
| MMP-10 | P09238 | Matrix metalloproteinase-10 |
| MCP-1 | P13500 | Monocyte chemotactic protein 1 |
| MCP-2 | P80075 | Monocyte chemotactic protein 2 |
| MCP-3 | P80098 | Monocyte chemotactic protein 3 |
| MCP-4 | Q99616 | Monocyte chemotactic protein 4 |
| CD244 | Q9BZW8 | Natural killer cell receptor 2B4 |
| NT-3 | P20783 | Neurotrophin-3 |
| NRTN | Q99748 | Neurturin |
| OSM | P13725 | Oncostatin-M |
| OPG | O00300 | Osteoprotegerin |
| PD-L1 | Q9NZQ7 | Programmed cell death 1 ligand 1 |
| EN-RAGE | P80511 | Protein S100-A12 |
| SLAMF1 | Q13291 | Signaling lymphocytic activation molecule |
| SIRT2 | Q8IXJ6 | SIR2-like protein 2 |
| STAMBP | O95630 | STAM-binding protein |
| SCF | P21583 | Stem cell factor |
| ST1A1 | P50225 | Sulfotransferase 1A1 |
| CD6 | P30203 | T cell surface glycoprotein CD6 isoform |
| CD5 | P06127 | T-cell surface glycoprotein CD5 |
| CD8A | P01732 | T-cell surface glycoprotein CD8 alpha chain |
| TSLP | Q969D9 | Thymic stromal lymphopoietin |
| TNFB | P01374 | TNF-beta |
| TRANCE | O14788 | TNF-related activation-induced cytokine |
| TRAIL | P50591 | TNF-related apoptosis-inducing ligand |
| TGF-alpha | P01135 | Transforming growth factor alpha |
| TWEAK | O43508 | Tumor necrosis factor (Ligand) superfamily, member 12 |
| TNF | P01375 | Tumor necrosis factor |
| TNFSF14 | O43557 | Tumor necrosis factor ligand superfamily member 14 |
| TNFRSF9 | Q07011 | Tumor necrosis factor receptor superfamily member 9 |
| uPA | P00749 | Urokinase-type plasminogen activator |
| VEGF-A | P15692 | Vascular endothelial growth factor A |

Table S2. AUC (95% CI) for DEPs alone or in combination with STAMBP comparing ASD to HC.

| Protein Symbol | Single-protein AUC (95% CI), Rank | STAMBP+DEP AUC (95% CI), Rank |
| --- | --- | --- |
| STAMBP | **0.7218 (0.5946 − 0.8489), 1** | — |
| ST1A1 | 0.7107 (0.5827 − 0.8387), 2 | 0.7268 (0.601 − 0.8526), 9 |
| SIRT2 | 0.7016 (0.5713 − 0.8319), 3 | 0.7329 (0.6091 − 0.8566), 8 |
| MMP-10 | 0.7006 (0.568 − 0.8332), 4 | **0.7681 (0.6496 − 0.8867), 1** |
| AXIN1 | 0.6794 (0.5459 − 0.8129), 8 | 0.7147 (0.5858 − 0.8436), 12 |
| CD40 | 0.6794 (0.5475 − 0.8114), 8 | 0.7228 (0.5955 − 0.85), 11 |
| IL-18R1 | 0.6925 (0.562 − 0.823), 6 | 0.7621 (0.6442 − 0.8799), 2 |
| CD244 | 0.6875 (0.5564 − 0.8186), 7 | 0.7339 (0.6081 − 0.8596), 7 |
| CXCL1 | 0.6935 (0.5598 − 0.8273), 5 | 0.7248 (0.598 − 0.8516), 10 |
| IL18 | 0.6532 (0.5159 − 0.7905), 11 | 0.7560 (0.6371 − 0.875), 3 |
| PD-L1 | 0.6734 (0.5402 − 0.8066), 9 | 0.7530 (0.6325 − 0.8735), 4 |
| CSF-1 | 0.6583 (0.5211 − 0.7954), 10 | 0.7450 (0.6228 − 0.8671), 5 |
| CST5 | 0.624 (0.4848 − 0.7632), 12 | 0.7389 (0.6149 − 0.8629), 6 |

AUC, the areas under the receiver operating characteristic curves; CI, confidence interval; DEP, differentially expressed proteins; ASD, autism spectrum disorder; HC, healthy control.

**
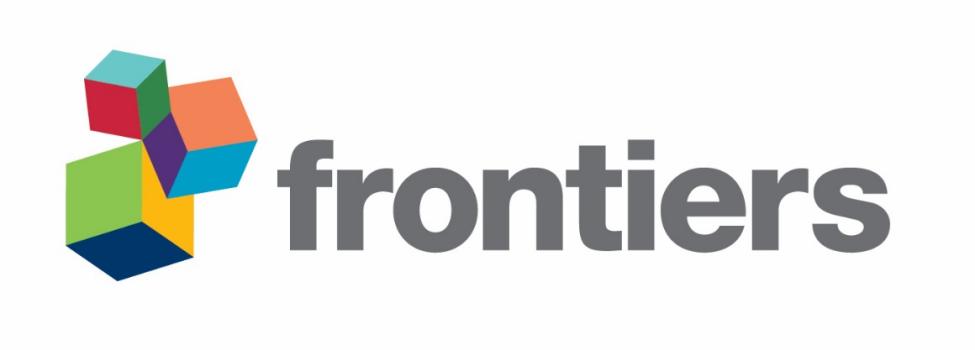
**
